# The COVID-19 Pandemic in a Paediatric Population: a Health Care Perspective

**DOI:** 10.34763/jmotherandchild.20212502.d-21-00008

**Published:** 2022-04-01

**Authors:** Viral P. Maru, Salil Bapat

**Affiliations:** 1 Department of Paediatric and Preventive Dentistry, Government Dental College and Hospital, Mumbai, India; 2 Department of Public Health Dentistry SMBT Dental College, Hospital and Research Center, Ghulevadi, Amrutnagar (Sangamner), India

**Keywords:** COVID-19, Paediatric population, Paediatric health care

## Abstract

SARS-CoV-2, a member of the coronaviridae family, has caused a deadly pandemic known as the Coronavirus disease 2019, (COVID-19). Paediatric patients get infected by direct exposures or airborne droplets, mostly from households with a cluster of infection or a history of exposure to outbreak areas. COVID-19 is contagious in children (those under the age of 18), with an average incubation time of around 6.5 days. Paediatric patients account for less than 5% of total COVID-19 patients worldwide, and hence studies involving such subjects are unlikely in the near future. As a result, there are inadequate data about COVID-19 infection in children. Therefore, we present this systematic review to understand the epidemiology, clinical features, diagnostic outcomes, and available options for the management of COVID-19 in paediatric populations in order to advise clinical and public health policy choices for safety of children worldwide.

## Introduction

**Figure 1:**
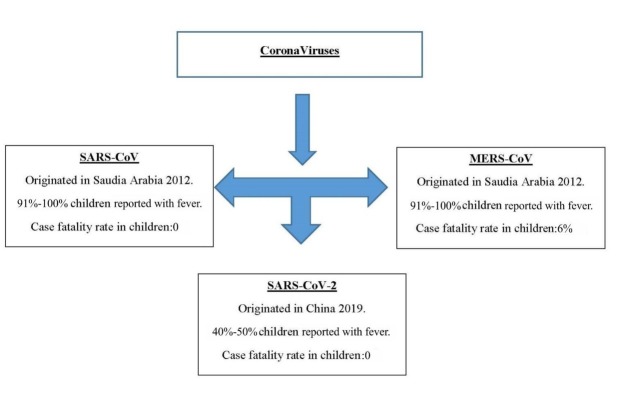
Evolution of Coronaviruses.

Coronaviruses (CoVs) are zoonotic RNA viruses that belong to the Coronaviridae family, which belongs to the Nidovirales order. These are single-stranded and encompassed viruses (Figure 1). [1] In humans, CoV infections may lead to abdominal or breathing issues, which may range from fever and flu to severe conditions like pneumonia, bronchitis, coagulopathy, and Severe Acute Respiratory Distress Syndrome (ARDS), leading to failure of multiple vital organs and death. [2,3,4,5,6] Human Coronaviruses (HCoVs) are linked to illnesses such as cystic fibrosis, emphysema, asthma, and chronic bronchitis, among others. [7,8] There are several classes of CoVs, namely alpha coronaviruses, beta coronaviruses (which are typically found in warm-blooded creatures like rodents, bats, civets, and even humans), gamma coronaviruses, and delta coronaviruses (which typically affect birds). [9,10] Humans are typically affected by four CoVs, namely HCoV2-229E, -NL63, -HKU1, and -OC43. The NL63 and 229E forms have evolved from bats, while 229E 19 and OC43 have emerged from dromedary camels and dairy cattle, respectively. There is currently no information regarding the evolution of the human coronavirus HCoV-HKU1.

Several coronaviruses are associated with warm-blooded creatures, especially bats; however, they haven’t been associated with humans. CoVs mutate and recombine rapidly, which allows them to be transmitted to humans. In 2002, the outbreak of the novel SARS-CoV in China was believed to have started with the virus being transmitted to humans from bats or civet felines. [11,12,13,14] The Middle East Respiratory Syndrome is caused by the MERS-CoV, which caused an outbreak in 2012 in Saudi Arabia, where dromedary camels were the carriers and the virus infected the human population. [15,16]

COVID-19 is a new beta coronavirus that belongs to the sarbecovirus β lineage. [17] Genome sequencing confirmed that COVID-19 is correlated to the SARS CoV (87% to 89% unique nucleotides), which is commonly carried by Chinese horseshoe bats (bat-SL-CoVZC45). The COVID-19 epidemic began in the Chinese city of Wuhan, in the province of Hubei. On December 31^st^ 2019, the Hubei Health Commission announced that several adults were suffering from pneumonia of unknown cause. A market selling fish and animals was thought to be the place where the outbreak started. The virus spread rapidly through symptomatic and asymptomatic human carriers, where physical contact and respiratory spatter led to transmission. Beginning with Wuhan, the SARS-CoV-2 virus spread to nearby areas and subsequently to other countries, thereby evolving into a pandemic. COVID-19 is the terminology used for the illness induced by the SARS-CoV-2 virus. [18]

Though the paediatric population is typically less prone to severe infection, the circumstances could change soon. The paediatric population is mostly asymptomatic; however, prolonged faecal shedding could lead to a higher transmission rate. On May 14^th^ 2021, there were 16.1 crore [18] COVID-19 positive cases globally, while India had 2.4 crore individuals suffering from COVID-19, as reported by the Ministry of Health and Family Welfare. [19] As of May 6^th^ 2021, over 3.85 million children had tested positive for COVID-19. [18] However, the severity of the illness is lower in children compared with that of the adult population. India reported the first case of vertical transmission of COVID-19 on July 28^th^ 2020, where the virus spread from mother to child, as reported by the Sassoon General Hospital in Pune. [19]

Currently, there is inadequate information about the prognosis for COVID-19 or its clinical and management aspects. In this context, the severity of the symptoms and their spectrum vary immensely between paediatric and adult populations. Hence, it is crucial to facilitate a simpler understanding of the manifestation and progression of COVID-19 in children. This systematic review is intended to explore the epidemiology, transmission, clinical manifestation, diagnostic aspect, and management of COVID-19 in children.

## Material and Methods

### Protocol registration

We followed the Preferred Reporting Items for Systematic Reviews and Meta-Analyses (PRISMA) statement guidelines. [20] The study protocol was registered at the PROSPERO database (https://www.crd.york.ac.uk/PROSPERO) under code CRD42021255406.

### Research question

What is the current state of knowledge in the literature regarding the epidemiology, transmission, clinical characteristics, diagnosis, and management of COVID-19 infection in the paediatric population?

### Search strategy

The present literature review was carried out using the following electronic databases: MEDLINE, EBSCOhost, and well known journals for all English-language papers related to children published from inception to April 30^th^ 2021 using search strategy (“coronavirus COVID-19, or COVID) and (babies, infants, preschool children, or paediatric patients).

The WHO COVID-19 database was searched using the terms children, paediatric, COVID-19, disease transmission, clinical features, diagnosis, treatment, and management, by manually trawling the collections for related titles.

### Inclusion criteria

Case reports, case series, and cross-sectional studies that provided the details on the epidemiology, transmission, clinical characteristics, diagnosis, management, and treatment options for COVID-19 in children and adolescents (aged < 21 years) met the inclusion criteria for this research. There were no restrictions on the kinds of experiments or the countries from which they came.

### Exclusion criteria

The current review rejected manuscripts that only reported on adults or other respiratory viruses, did not apply to the clinical or epidemiological areas of interest, and were not published in English.

### Study selection and data extraction

Given the inclusion and exclusion criteria, two reviewers (MV, BS) independently screened the title and abstract of the selected manuscripts. The reviewers read the studies independently and extracted the data using a data extraction form created specifically for this review. The following information was included on this form: the author, the title of the paper, the type of study, purpose, and the results. Any differences between the two reviewers were settled by discussion.

### Quality assessment of studies included

The information was collected from selected studies and tabulated using the standardized data extraction tools kit by JBI. The JBI Data Extraction Forms were utilized following the study design. [21] Data extracted from cross-sectional studies included specific details about inclusion in the sample, measurement about the exposure, standardized criteria used for measurement, confounding factors, and reliability of the outcomes measured and appropriateness of the statistical tests used. Similarly, data extracted for case series and case reports included demographic characteristics, clinical conditions, assessment methods, and post-intervention clinical conditions. [22] To reduce the possibility of errors, all the results were subjected to double data entry.

## Results

**Figure 2:**
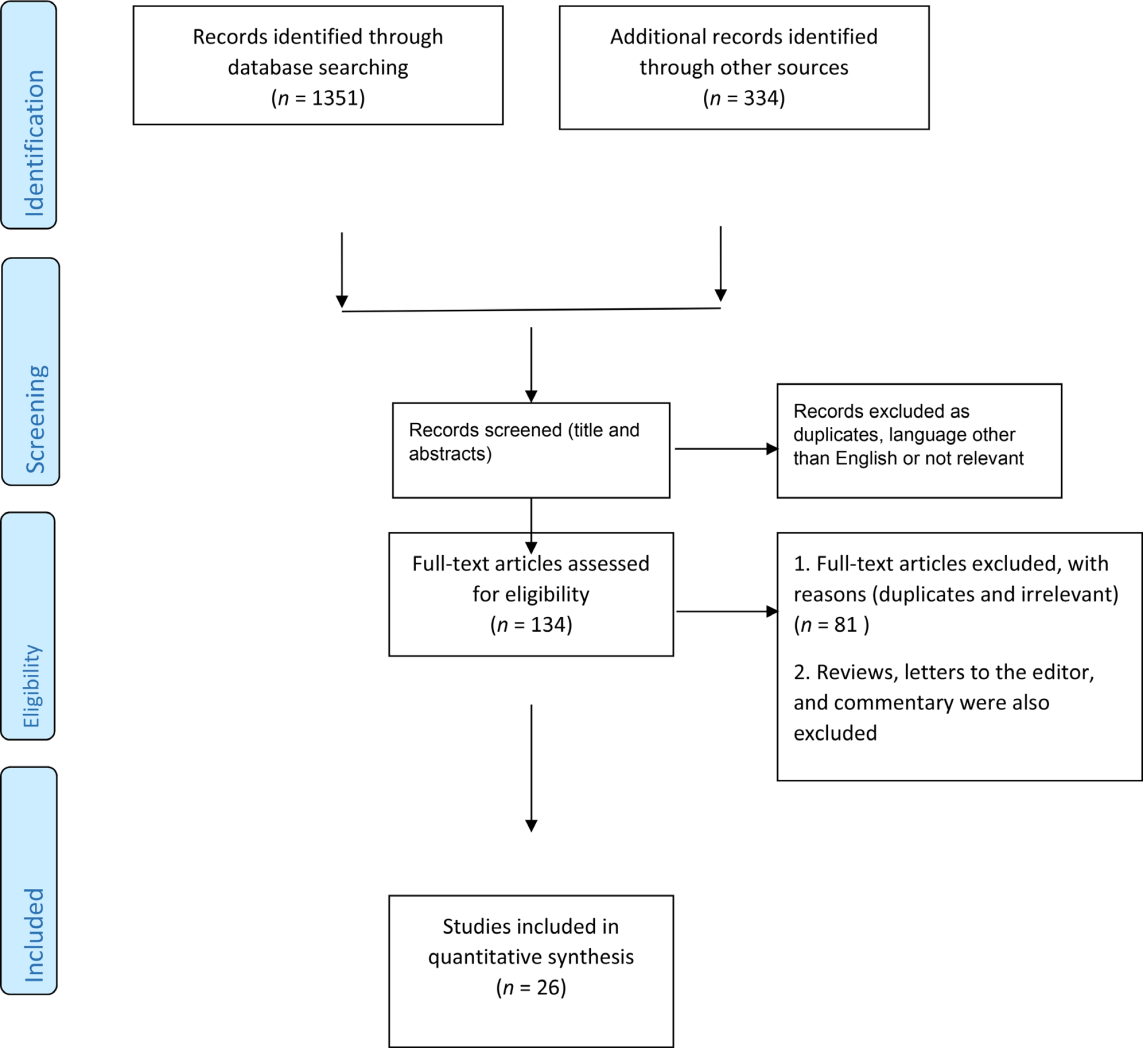
PRISMA flow diagram.

The electronic databases and hand search for the WHO COVID-19 literature database yielded 1,351 and 334 reports respectively. Out of a total of 1,685 studies, 1,551 studies were excluded due to duplication, a language other than English, non-availability of full text and irrelevant articles. In all, 134 studies with full text were assessed for eligibility. Out of 134 studies assessed, further 81 studies were excluded. Finally, 26 manuscripts were analyzed in the present review. The paper retrieval technique and the papers found using a mix of key terms are shown in Figure 2. Due to the variability in the methods of diverse studies, we did not do a meta-analysis.

### Characteristics of studies included

Table I summarizes the characteristics of the selected literature concerning epidemiology, clinical manifestation, transmission, diagnosis, and treatment options of COVID-19 in the paediatric population.

**Table 1:** Characteristics of the studies included.

Sl No.	Author’s name	Title of study	Type of study	Purpose of study	Results of study
1.	Chuming C et al.^23^	Coronavirus Disease- 19 Among Children Outside Wuhan, China	Cross- sectional. (31 children < 18 yrs.)	Description of characteristic features of infection in children.	12 infants (38.7%) showed remaining two-thirds had minor cases in children). The majority not have any underlying illnesses had underlying diseases, one and the other had duplicate the children were in familial
2.	Feng K et al.^24^	Analysis of CT features of 15 Children with coronavirus 2019 novel infection.	Cross sectional. (15 children of age 4–14 yrs.)	To study computed tomography features in children with COVID-19.	For their first chest CT images, lesions, while 9 patients had lesions. Seven cases of small opacities and 2 cases of opacities were found.
3.	Pan X et al.^25^	Asymptomatic cases in a family cluster with SARS-CoV-2 infection	Case series.	Case study of clinical characteristics family cluster of COVID-19 (mother, 33; father, 35; 3-year-old boy).	▪ Clinical Manifestation: asymptomatic.
4.	Sun K et al.^26^	Early epidemiological analysis of the coronavirus disease 2019 outbreak based on crowdsourced data: a population-level observational study.	Cross- sectional study. (9,507 children under the age of 15 yrs.)	A population-level research analysis is being conducted using data from a health care-oriented social network that is currently	▪ Clinical manifestation: no
				broadcasting COVID-19 news stories from local and national Chinese health authorities and agencies with regards to COVID-19 epidemiology patterns, including the spread of the epidemic in China.	
5.	Wang P et al.^27^	Epidemiological characteristics of 1212 COVID-19 patients in Henan, China	Cross- sectional study.	Cross-sectional analysis publicly of available data.	Out of 1,212 COVID-19 cases aged 0–10 years (1.98%) and 21 cases aged
6.	Henry BM et al.^28^	Preliminary epidemiological analysis on children and adolescents with novel coronavirus disease 2019 outside Hubei Province, China: an observational study utilizing crowdsourced data	Cross- sectional study. (children < 19 yrs) 53 children (0–12 yrs) and 27 adolescents (13–19 yrs).	To study crowdsourced data outside of Hubei province (i.e., includes mainland China minus Hubei and rest of the world).	Fever was the most common followed by cough (36%). asymptomatic. A total of 29 noted to have an infected family.
7.	Yu H et al.^29^	Data-driven discovery of clinical routes for severity detection of COVID-19 paediatric cases	Cross- sectional study.	To assess 105 cases of COVID-19 infected children diagnosed in Wuhan. 64 males, 41 females.	Shortness of breath, supported respiration, apnea, cyanosis, dehydration, and progressive increase of lactate were reported. 8 were critically ill.
8.	Tank A et al.^30^	A retrospective study of the clinical characteristics of COVID-19 infection in 26 children.	Case series.	To assess clinical features in children with COVID-19.	▪ 9 patients were asymptomatic. Fever was the most common symptom. Other symptoms, cough (in eleven of seventeen patients), rhinorrhea (in two), diarrhea (in two), vomiting (in two), were also observed. A small minority of patients had lymphocytopenia. ▪ According to chest CT scan, 11 patients showed unilateral pneumonia, 8 patients had no pulmonary infiltration.
9.	Li-Na Ji et al.^31^	two family clusters		features in	▪ Mild respiratory or gastrointestinal
		cases.	Case series.	children COVID-19. with	Both of them had normal ▪
				(2 children)	▪ After general and symptomatic children recovered quickly.
10.	Park JY et al.^32^	First Paediatric Case of Coronavirus Disease 2019 in Korea.	Case report. (10 yr old girl)	Reporting the features of first paediatric patient with COVID-19 in Korea.	The girl reported with low computed tomography (CT) nodular consolidations with opacities in subpleural areas She recovered without any
	Rahimzadeh			Reporting	▪ All children had at least one
11.	G et al.^33^	COVID-19 infection in Iranian Children: A	Case series.	features of	▪ Fever, chills, myalgia, cough, and crackle were common
		case series of 9 patients.	(9 children with age 2–	COVID-19 in Iranian children.	patients presented with diarrhea, nose. Three cases had leukopenia
			10 yrs).	▪	All cases received supportive therapy.
12	Xia W et al.^34^	Clinical and CT features in paediatric patients with COVID‐ 19 infection: Different points from adults	Cross- sectional study.	Case series of 20 paediatric patients from Wuhan Children's Hospital who presented from January 23 to February 8, 2020, with the primary goal of describing radiological characteristics.	Average length of hospital confirmed by pharyngeal swab acid test. Seven of 20 had underlying (congenital). No evidence that the virus. Thirteen of 20 cases with COVID-19
13.	Xu H et al.^35^	A follow-up study of children infected with SARS-CoV-2 from Western China.	Cross- sectional study	To study the features and the duration of positive nucleic	▪ 11 children (34%) were asymptomatic, whom 6 had normal computed scan images.
			(32 children).	acid in children with COVID-19.	▪ All children recovered. No
14.	Shekerdemian L et al.^36^	Characteristics and Outcomes With Coronavirus of Children Disease 2019 (COVID- 19) Infection Admitted	Cross- sectional study,	To experience assess the of COVID-19 in paediatric	▪ 40 children (83%) had preexisting medical conditions, 35 (73respiratory symptoms, and invasive ventilation.
		to US and Canadian Paediatric Intensive Care Units.	(48 children).	intensive care units.	▪ The hospital mortality rate
15.	Dong Y et al.^37^	Epidemiology of COVID-19 among children in China	Cross- sectional study. (2,135	To study the epidemiological and transmission	▪ More than 90% of all children mild, or moderate cases. illness onset to diagnoses days).
			children with mean age 7 yrs.)	patterns in children with COVID-19	▪ Mild 314 (43.1); Moderate (2.5); Critical 3 (0.4); Missing (0.1).
16.	Qui H et al.^38^	Clinical and epidemiological features of 36 children with coronavirus disease 2019 (COVID- 19) in Zhejiang, China: an observational cohort study.	Cross-sectional study. (36 children with mean age 8.3 years).	To assess the clinical and epidemiological features of children with COVID-19.	▪ Transmission: Through close members (32 [89%]) or a epidemic area (12 [33%]); both exposures. ▪ Clinical manifestation: 19 pneumonia; 17 (47%) had either were asymptomatic upper respiratory symptoms symptoms: fever (36%) and those with fever, four (11of 38.5°C or higher, and temperature of 37.5°–38.
					▪ Management: All children by aerosolization twice a lopinavir–ritonavir syrup needed oxygen inhalation.
17.	Qiu C et al.^39^	Transmission and clinical characteristics of coronavirus disease 2019 in 104 outside- Wuhan patients, China.	Cross-sectional study. (3 children)	Contact investigation was conducted on each patient who was admitted to the assigned	Transmission through family
				hospitals in Hunan Province.	
18.	Wang S et al.^40^	A Case Report of Neonatal 2019 Coronavirus Disease in China.	Case report.	Reporting a case of neonatal COVID-19 infection in China with pharyngeal swabs tested positive by RT-PCR assay, 36 hours after birth.	The infant was isolated in hospital. x-ray showed thickened abnormalities in heart and palate.
19.	Kam KQ et al^41^	A well infant with Coronavirus Disease 2019 (COVID-19) with high viral load.	Case report. (6-month-old infant).	A case report highlighting the difficulties in establishing the true incidence of COVID-19.	Infant was asymptomatic. Diagnosis through the presence stools of infant.
		A Case Series of			▪ Eight (80%) children had patients with COVID-19 of travel to Wuhan or Wuhan.
		Children With 2019 Novel Coronavirus Infection: Clinical and	Case series. (10 children	To study clinical and epidemiological	▪ For the 3-month-old infant, symptomatic COVID-19after the sick baby without
20.	Cai J et al.^42^	Epidemiological Features	with mean 74 months of age)	features in children with COVID-19.	▪ The interval between symptom index symptomatic case (mean: 6.5 days) and the onset and departure from and 9 days.
					▪ Eight (80%) patients had 4 (40%) had sore throat, 3 2 (20%) had sneezing and
					▪ Chest radiograph revealed in 4 (40%) of 10 patients.
					▪ Out of those admitted symptomatic. Fever was from chills, cough, loss shortness of breath etc.
21.	Graff K et al.^43^	Risk factors for severe COVID-19 in children.	Cross- sectional study 454 children. 191 females.	To describe the epidemiology and risk factors for severe COVID-19	▪ 45% of children with COVID comorbid condition. The comorbid conditions identified (16.7%), gastrointestinal disease (10.6%).
			Average age 11 years.	disease in children.	▪ Diagnosis through nasopharyngeal washes/aspirates, and bronchoalveolar and RT PCR.
					▪ 55% required respiratory critical care.
		COVID-19-related giant coronary aneurysms in an infant		Reporting a case	
22.	Ghatasheh G et al.^44^	with multisystem inflammatory disorder in children: the first	Case report (infant of 9 months of	of COVID-19 related aneurysms in an	▪ The clinical and biochemical compatible with complicated disease (KD).
		case report from the united arab emirates and the arab region.	age)	infant MID in children.	▪ Treated with intravenous anticoagulation for 3 weeks.
23.	Sethuraman U et al.^45^	Multisystem inflammatory syndrome in children	Case series.	To study the clinical features of MIS in	▪ Most of them were African older than 5 years old who once in the previous 48
		associated with novel coronavirus SARS-		children with COVID-19.	▪ More than a third of the with asthma being the most
		CoV-2: Presentations to a paediatric emergency department in Michigan.			▪ The most frequent signs gastrointestinal symptoms or diarrhea), anorexia, symptoms were uncommon.
		Comparison of clinical			▪ Although 44.4 percent abdominal tenderness, only right lower quadrant tenderness.
		and epidemiologic characteristics of young febrile infants	Cross	To evaluate and compare clinical	▪ In 2020, fewer febrile infection or a positive respiratory in previous years (6 percent
	Leibowitz J et	with and without	sectional study.	and epidemiological	▪ respectively). COVID-19 was the most
24	al.^46^	Severe Acute	(124 infants	features in febrile	in 2020, with 20 out of
		Respiratory Syndrome Coronavirus-2 Infection.	of age < 57 days).	infants with or without COVID -	▪ Infants infected with COVID-to register as Hispanic.
				19.	▪ None of the babies who substantial bacterial infection.
		Severe acute respiratory syndrome coronavirus 2 clinical	Cross	Review of	▪ Overall, 143 (51%) had respiratory (25%) had MIS-C, and 69 manifestations including fever.
25	Fernandes DM et al.^47^	syndromes and predictors of disease severity in hospitalized children and youth.	sectional study (281 paediatric patients).	clinical features of 281 hospitalized kids with COVID-19.	▪ Patients with MIS-C were non-Hispanic black compared respiratory disease (35% (2%) died and 114 (41%) intensive care unit.
					▪ Race/ethnicity or socioeconomic predictive of disease severity.
26	Simpson JN et al.^48^	Results of testing children for severe acute respiratory syndrome coronavirus-	Retrospective cross- sectional study.	To study the demographics, clinical features, and test results of	▪ 28.2% had a positive polymerase test.
		2 through a community-based testing site.	(1,445 children < 21 years of age)	children with COVID-19 in community	▪ The daily positivity rate increased period, from 5.4% during 47.4%.
				setting.	▪ Patients with fever or cough contact with someone with infection were more likely

#### Epidemiology

During the COVID-19 pandemic, transmission from individuals has been significant. Virus incubation time and serial interval time are understood to be five to six days and eight days, respectively. These numbers are similar to the SARS and MERS outbreaks. The case fatality for COVID-19 is understood to be in the range of 0.9% to 3% [49], which is significantly less compared with SARS-CoV and MERS-COV, which ranged from 6% to 17% and 20% to 40%, respectively.

Several studies suggested that younger individuals, especially the paediatric population, have a milder severity of infection compared with relatively older individuals. COVID-19–positive paediatric population has been mostly asymptomatic. Moreover, the percentage of the paediatric population having a mild or moderate illness is 5.9%, which is significantly less compared with that of the adults, for which the corresponding percentage is 18.5%. Probable causes for younger individuals having lower infection rates and severity could be limited virus exposure, higher seclusion by staying at home, and relatively less exposure to cigarette smoke and pollution (thereby leading to better respiratory health). A diminished immune response is one factor that renders senior citizens more vulnerable to COVID-19. Interaction between infected individuals could potentially trigger lesser replication of the virions due to rivalry. Additionally, the working, expression, and other aspects of viral receptors, such as Angiotensin-Converting Enzyme-2 (ACE2) could be the determinants of age-specific vulnerability to severe forms of COVID-19 infection. [27,50,51,52]

Relatively fewer cases of severe COVID-19 illness in children are a challenge to precisely determining the clinical aspects of illness, which is compounded by an absence of biomarkers to ascertain disease severity. [53] Dong Y et al. [37] examined 2,143 children, of whom 13% were determined to be COVID-19 positive were asymptomatic. Epidemiological extrapolation is challenging since the likelihood of asymptomatic individuals being tested is lower, which is one reason that potentially promotes the spread of the disease. Children could also be suffering from other infections, which may render the detection of COVID-19 inconsequential from a clinical standpoint. The COVID-19 infection prognosis could be worsened by weight gain and cigarette smoke exposure. [53]

#### Transmission

COVID-19 infection originated from a wet market in Wuhan, China, having passed from a bat to a person via an intermediary host, such as a pangolin. The most common mode of human-to-human transmission is direct or indirect contact with virus-laden respiratory droplets circulated by infected persons while sneezing or coughing.

COVID-19 has a similar mode of transmission, in which a person comes into contact with fomites, such as an infected patient's clothes or hands, dining utensils, door handles, counter surfaces, and other objects touched, soiled, or used by the infected person, causing respiratory droplets to be spread. SARS-CoV-2 is not thought to be able to penetrate the keratin layer of healthy human skin, but it is thought to enter the body through mucosal surfaces when contaminated hands come into contact with the eyes, nose, or mouth.

According to observations, it is expected that C0VID-19–positive paediatric populations are secondary cases infected through contact with any COVID-19 positive adult. There is a major scope for intra-family spread. [42] It is yet to be found whether COVID-19 infection can be transmitted through contact with infected stools. [54–55] According to a study, 80% of children who were COVID-19 positive per tests done through nasopharyngeal swabs continued to show repeat positive results for Real-Time Reverse Transcriptase-Polymerase Chain Reaction (RT-PCR) of rectal swabs, even after their nasopharyngeal swabs turned out to be negative. [36] However, it cannot be claimed with certainty that the identification of virus by RT-PCR in faecal waste characterized by viral genomic components or lively viral replication is a characteristic symptom for COVID-19. However, it does imply that viral shedding from the stomach lasts longer and is of more significance than that from the lungs.

#### Clinical features in children

Three series of COVID-19 cases were reported for paediatric patients in Wuhan, where 171, 20, and 10 patients became symptomatic after an incubation period ranging from 2 to 14 days. [34,42,56] Positive diagnosis was made using the RT-PCR test on 171 children, where the reported symptoms were dry cough (48.5%), sore throat (46.2%), fever (41.5%), tachypnoea (28.7%), diarrhoea (8.8%), runny nose (7.6%), tiredness (7.6%), and vomiting (6.4%). This study revealed that 15.8% of the paediatric patients were asymptomatic, while 19.3% and 64.9% of the patients were found to have disorders of the upper respiratory tract, and pneumonia, respectively. [42]

In an evaluation of 2,143 paediatric individuals, 34% had COVID-19, which was confirmed through laboratory tests, while the remaining individuals were only suspected to be ill. [37] Among the paediatric patients, 12.9% were asymptomatic. On the other hand, 43.1% of the patients had mild illness compared with 41% who were moderately ill. Of the patient pool, 2.5% had a severe illness, while 0.4% were critical. Paediatric subjects having a confirmed COVID-19 diagnosis exhibited slower progression compared with the suspected population, which indicates that the illness caused by COVID-19 could be less severe compared with other respiratory disorders. The minimum time between infection onset and the confirmed diagnosis was 48 hours. For paediatric patients having a severe or critical form of the illness, the incidence was 10.6% for infants less than 12 months of age, while it was 7.3%, 4.2%, and 4.1% for children of 1–5 years, 6–10 years, and 11–15 years, respectively. Individuals over 15 years of age had an incidence of 3.0%. These numbers suggest that infants are more susceptible to COVID-19 infection when compared with older children. The individuals considered as having a severe form of the illness had complaints of hypoperfusion and hypoxemia after seven days of onset. Common symptoms of COVID-19 in the paediatric population include liver dysfunction, septic shock, myocarditis, disseminated intravascular coagulation, Acute Respiratory Distress Syndrome (ARDS), and acute kidney injury. [57] In the context of Chinese paediatric patients, the predicted mortality rate was 0% for children up to 9 years of age, while for those of 10–19 years of age, the mortality rate was 0.18%, both of which are very low compared with the 2.3% for adults in the 70–79 years age bracket. [49]

The oral cavity is the first interaction point between the human body and the external environment. Viral colonization of the oral cavity has a significant role in catching a viral infection. The mucosa of the tongue has a high expression of ACE-2.

In the context of COVID-19 infection, oral health is affected indirectly, though it is multi-directional and manifests itself as an immune response. Acute COVID-19 infection and the associated treatment may potentially affect oral health due to the highly susceptible oral mucosa and the compromised immune response of the body. It is crucial to know that the cytokine storm created as a result of the dysregulation of humoral and cellular processes could lead to autoimmune disorders in the oro-pharyngeal region. Health deterioration due to infection and side effects of treatment could lead to oral lesions by way of opportunistic infection.

Decreased ability to taste is a common symptom of COVID-19. The changes in taste could be attributed to the viral interaction with the ACE-2 receptors through which the virions enter the cells. The epithelial cells of the oral mucosa have a strong presence of ACE-2 receptors. Other oral symptoms include xerostomia and chemosensory disturbance. Paediatric patients are reported to have ulcers in the oral cavity due to COVID-19; however, the pathogenesis remains unknown. Therefore, it is crucial to investigate oral alterations using oral examination on COVID-19–positive paediatric patients. [58–59]

#### Diagnosis of COVID-19 in children

Nasopharyngeal RT-PCR tests and the throat swab tests are proposed as diagnostic tests for determining the presence of COVID-19 nucleic acid, which confirms COVID-19 infection. [28] Samples for RT-PCR testing may also be obtained by using bronchoalveolar lavage and endotracheal secretions.

The paediatric population has lesser lymphocytopenia and an increase in inflammatory markers compared with COVID-19–positive adults; however, there are inadequate data to ascertain the reasons for such observations. Henry BM and team [28] evaluated 12 studies and reported that 69.2% of COVID-19 positive children had normal leucocyte levels. On the other hand, 6% had reduced neutrophils, 4.6% had increased neutrophils, and 3% of the population had reduced lymphocytes. Among paediatric patients, about 13.6% were observed to have elevated C-reactive protein (CRP) levels, while 10.6% had higher levels of procalcitonin. Therefore, lymphocytes, CRP, and procalcitonin are important markers of severe COVID-19 illness and their levels must be ascertained to make a positive diagnosis of COVID-19 and other infections.

While chest x-rays are nonspecific for the paediatric population, those suffering from a mild form of COVID-19 should not be subjected to Computed Tomography (CT) of the chest because of the risks of radiation exposure. [60] CT scans reveal ground-glass opacity for about one-third of the paediatric population. There have also been reports of peripherally distributed lung lesions with multilobar involvement **[**61] COVID-19 positive children exhibit consolidation with an associated halo, as observed on the CT images. It is challenging for clinicians to use chest CT images as conclusive evidence of COVID-19 infection since several illnesses have similar radiological indications. Plasma levels of IL-2, IL-7, and IL-10 are shown to be elevated in paediatric patients with serious COVID-19 disease. In addition, levels of interferon-gamma-inducible protein 10 (IP10), granulocyte colony-stimulating factor (GCSF), tumour necrosis factor (TNF), alpha monocyte chemoattractant protein 1 (MCP1), and macrophage inflammatory protein 1 alpha (MIP1A) are higher in COVID-19 patients.

#### Management and supervision of paediatric patients with COVID-19

Until now, there has been no concrete proof of the efficacy and safety of particular medications against COVID-19. In one case study, 36 children were given interferon twice a day via aerosol and 14 of them were also given lopinavir-ritonavir syrup. It was impossible to infer the influence of the antiviral drug since this was an observational trial with no monitoring arm and all of the children had mild-to-moderately severe disease. [38] Various antivirals and immunomodulatory medications, such as chloroquine, azithromycin, remdesivir, tocilizumab, and convalescent plasma therapy, were attempted in a recent report on children admitted to the ICU with COVID-19. [36]

In the initial course of infection, antiviral therapy is expected to be successful. Since there is currently insufficient evidence, immunomodulatory therapy may be recommended only where there is strong evidence of hyperinflammation or in the second step of the disease. On a case-by-case basis, compassionate off-label care can be offered. Off-label treatment of antivirals, immunomodulatory agents, immunoglobulins, and even convalescent plasma therapy have been reported anecdotally. [36,38]

### Quality of the studies included

**Figure 3:**
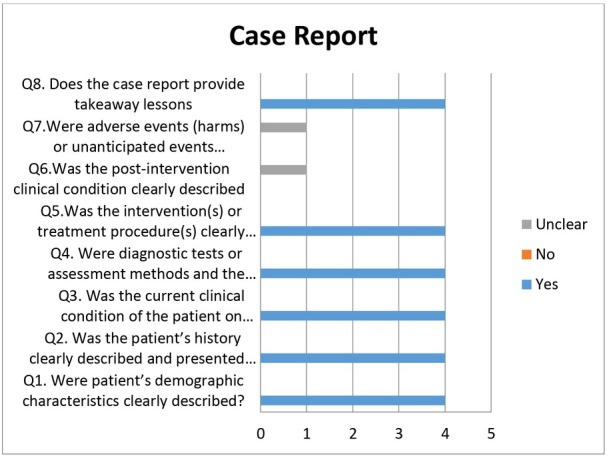
Quality assessment of case reports.

**Figure 4:**
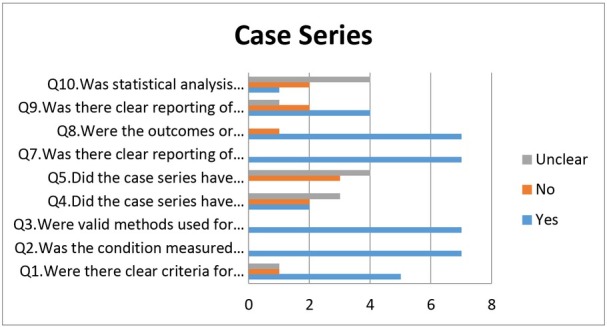
Quality assessment of case series.

**Figure 5:**
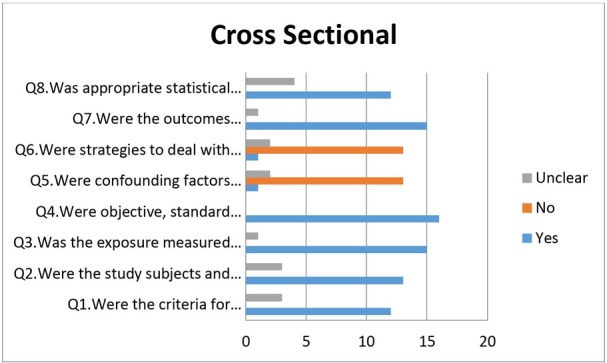
Quality assessment of cross-sectional studies.

Out of four case reports selected, three case reports showed low risk in all criteria, i.e., demographic characteristics, transmission, the clinical condition of the patient, diagnostic tests or methods, and intervention(s) or treatment procedure. However, one case report by Park JY et al. [32] showed moderate risk while reporting post-intervention clinical condition (Figure 3). Out of the six-case series included in the review, none of the studies fulfilled all the required criteria. All the studies showed low risk in the following criteria: (1) following a standard and reliable way to measure the condition, (2) use of a valid method for identification of the condition, (3) clear reporting of clinical information, and (4) clear outcomes or follow up results (Figure 4). Cross-sectional studies showed high risk in the identification of confounding factors; only one study by Graff K et al. [43] had identified confounding factors, whereas the other criteria, such as those for inclusion in the sample, study subjects and the setting, exposure measured, and standardized criteria used for measurement of the condition showed low risk (Figure 5).

## Discussion

Since the beginning of the pandemic, global understanding of COVID-19 epidemiology, clinicalfeatures, transmission, and management has continued to develop. Children have been shown to have lower rates of chronic disease and death than adults. The bulk of the results have come from China and may be skewed by ascertainment prejudice, which might be due to a larger-than-known number of milder instances among seniors and adolescents, or systematic ascertainment and testing bias toward elders with significant respiratory symptoms and diagnostic limitations. The bulk of the publications investigated have not been peer-reviewed.

Small case series and case studies have provided reliable information regarding the therapeutic characteristics and effects of COVID-19 in infants. In a case series of 105 children in Wuhan, reports of relatively moderate symptoms and some instances of silent infections emerged as key emerging patterns from the literature, with some evidence of severe cases (7.6%). On rare occasions, juvenile fatalities have been confirmed. [62]

There were no comprehensive reports of COVID-19 transmission from children that we could find. Many of the cases in infancy stem from maternal clusters, with children detected by interaction. Just one instance has been recorded in which a three-month-old child possibly became infected from her parents. [42] Mild and asymptomatic infections in infants and a high incidence of chest radiographic abnormalities have been reported. [42] To explain the length, persistence, and functional deficit associated with these results, longitudinal evidence would be needed.

**Figure 6:**
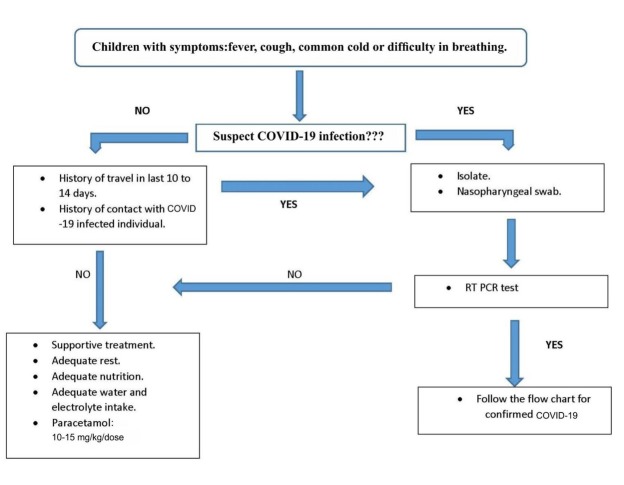
Proposed flow chart for management of children with COVID-19 like symptoms.

**Figure 7:**
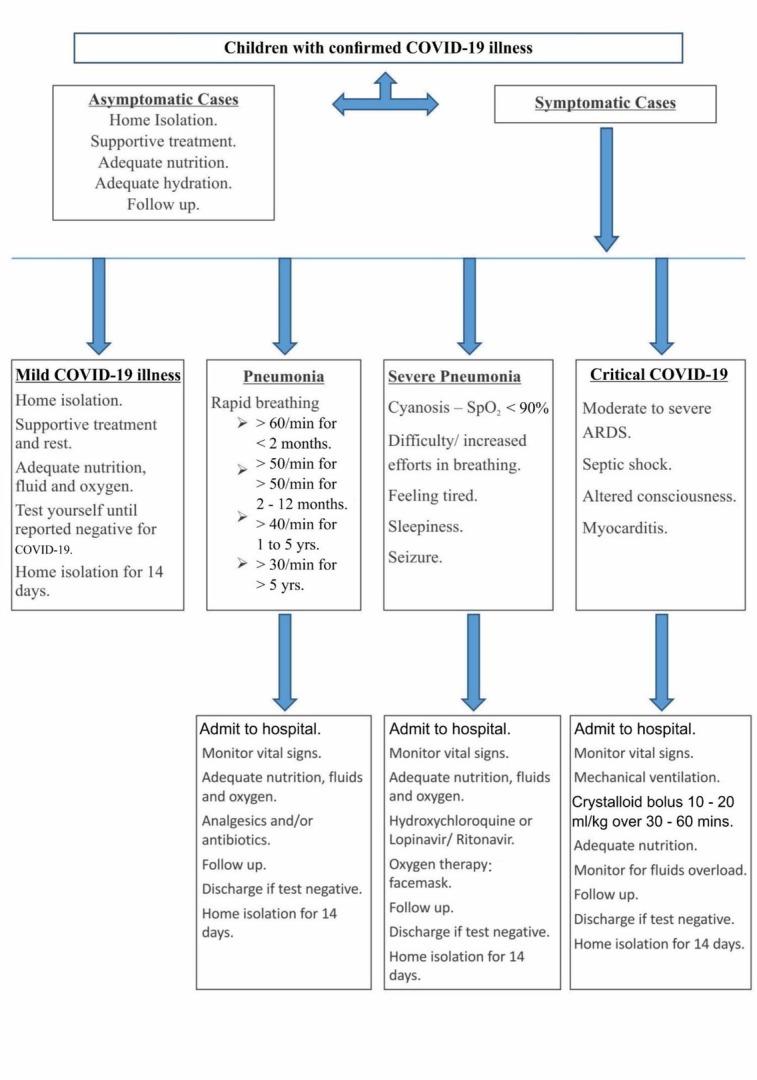
Proposed flow chart for management and supervision of COVID-19 positive children.

**Figure 8:**
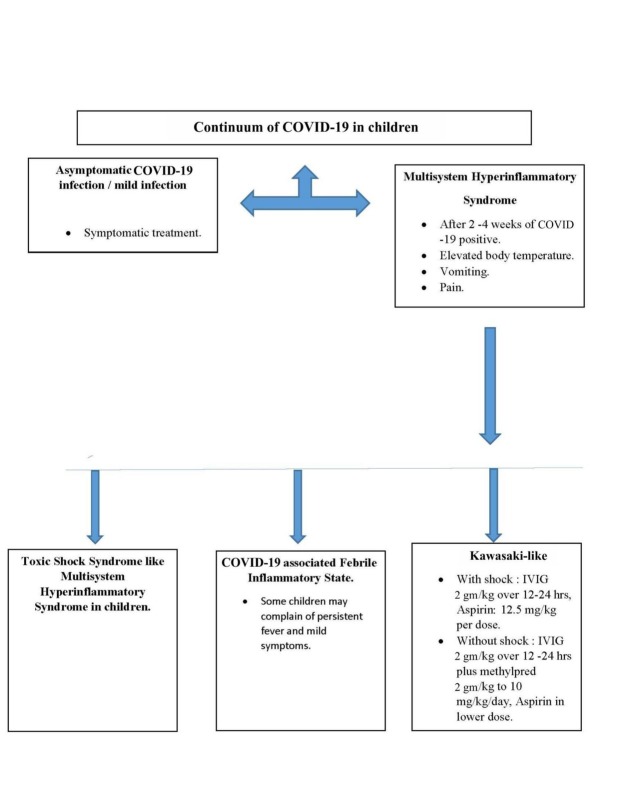
Continuum of COVID-19 in children.

Asymptomatic COVID-19 subjects are managed through supportive therapy, where aspects like calorie intake, nutrition, fluid and electrolyte levels, and oxygen intake are managed (Figure 6) Medical personnel should ensure that they have appropriate communication with the patients to help deal with any anxiety during disease management. PubMed literature has no reports of randomized clinical trials that suggest the treatment protocol for children having severe COVID-19 illness, ARDS, pneumonia, or shock. This could perhaps be the reason that the WHO has not provided guidelines specific to the treatment of severe COVID-19 cases involving children. Nevertheless, there is a definite need for research concerning the management and treatment of severe COVID-19 illness in the paediatric population (Figures 7 and 8). The inability to establish a standard protocol may lead to uncertainty regarding the prescription of antiviral or immunomodulatory therapy for children suffering from a severe form of COVID-19. Children considered to be at high risk, those exhibiting comorbidities, and those who have severe pneumonia should be taken care of by admission to the intensive care unit.

COVID-19 neonatal management in the case of new mothers who are infected involves a setup adjacent to the delivery room, with an isolated room for neonatal resuscitation. The social distancing of at least two meters is maintained between the newborn and the infected mother. Essential trained personnel wearing PPE are expected to be present in the delivery room to guide the infected mother on hand hygiene etiquette and how to wear a mask. A self-inflating bag and mask are generally used to provide positive pressure ventilation. To provide an intensive care facility to the newborn, a single patient room with negative pressure is used. The newborn is required to undergo testing after 24 hours of life followed by the second round of testing after 48 hours. The newborn needs to be tested after every 48– 72 hours till two successive negative test results are found. It is advised not to administer intravenous immunoglobulins or antivirals on newborns. The infected mother is allowed to breastfeed the newborn after all the precautionary measures are followed. [63]

As stated by Antiga L and associates [64], immuno-compromised newborns are not at a higher risk of severe COVID-19 because the primary driver of lung disease is the host's innate immune response. [47] A PubMed literature search does not result in any substantial theory or data on the relationship between severity of COVID-19 and infants with HIV or cardiac dysfunction or malnutrition.

Several pharmaceutical companies have successfully carried out trials and developed the vaccine against COVID-19 infection. The principal antigens for the disease are the structural spike glycoprotein “S” or its Receptor Binding Domain. The CoVs are characterized by their ability to undergo fast recombination and mutation, which in turn imposes a major challenge in the preparation of the vaccine. In the present pandemic, existing data and information can be reviewed regularly to help control the crisis and reduce viral spread, improve diagnosis and clinical suspicion, and protect the paediatric population worldwide.

The main drawback of the present review was that most of the studies were from China. For a better understanding of COVID-19 infection in children, research with a wider regional focus should be included. Data regarding transmission were inadequate. The management of COVID-19 infection in children was not specific. However, the data in this review offer an initial summary of the epidemiology, clinical features, transmission, and management of COVID-19 infection in children.

## Conclusion

This systematic review focused on COVID-19's epidemiology, clinical manifestations, transmission and management, which could help clinicians all over the world, particularly those in developing countries, with the onset of a new virus. Physicians will be able to keep track of patients, put control mechanisms in place, and save the situation from getting worse if they could detect the virus early and thus prevent it from spreading further. More detailed studies are required to assess the long-term prognosis of COVID-19 infection in the paediatric population worldwide.

## Legends

Tables:

- Table 1. Characteristics of the studies included.

Figures:

- Figure 1. Evolution of CoronaViruses.
- Figure 2. PRISMA flow diagram.
- Figure 3. Quality assessment of case reports.
- Figure 4. Quality assessment of case series.
- Figure 5. Quality assessment of cross-sectional studies.
- Figure 6. Proposed flow chart for management of children with COVID-19 like symptoms.
- Figure 7. Proposed flow chart for management and supervision of COVID-19 positive children.
- Figure 8. Continuum of COVID-19 in children
